# Preeclampsia: A Possible Complication of Primary Hyperparathyroidism

**DOI:** 10.1155/2016/7501263

**Published:** 2016-06-02

**Authors:** Bader Abdullah Alharbi, Mohammed Ali Alqahtani, Mohammed Hmoud, Essam Awadh Alhejaili, Reema Badros

**Affiliations:** ^1^King Abdullah International Medical Research Center, King Saud bin Abdulaziz University for Health Sciences, P.O. Box 9515, Jeddah 21423, Saudi Arabia; ^2^Department of Obstetrics and Gynecology, King Khaled National Guard Hospital, National Guard Health Affairs, P.O. Box 9515, Jeddah 21423, Saudi Arabia

## Abstract

*Background*. Primary hyperparathyroidism is rare in pregnancy. An association between primary hyperparathyroidism and preeclampsia has been reported in few cases worldwide.* Case*. A 28-year-old woman (gravida 2, para 0, and abortus 1) in her 27th week of gestation was hospitalized due to a high reading of blood pressure (194/115 mmHg) that was not accompanied by any symptoms or signs of preeclampsia. Incidentally, she was found to have a high adjusted calcium and serum parathyroid hormone (PTH) level during admission. Ultrasonographic examination of the neck revealed the presence of parathyroid adenoma. She was scheduled for surgical excision after receiving an intravenous hydration. Fetal ultrasonography revealed a growth restricted fetus with normal biophysical profile. On the sixth day of hospitalization, the patient complained of headache and epigastric pain, with elevated BP and proteinuria. The fetal nonstress test was “nonreassuring.” Subsequently, she had an emergency cesarean delivery and surgical removal of the adenoma. The mother and her newborn were then transferred to intensive care, where their clinical course was unremarkable. The mother was discharged after 3 days, while the neonate stayed for close observation for 60 days.* Conclusion*. Early recognition of primary hyperparathyroidism among women with preeclampsia is important to prevent maternal and fetal morbidity and mortality.

## 1. Introduction

Primary hyperparathyroidism is considered a relatively common endocrine disorder. It is the third most common endocrine disorder with a prevalence of 0.1–0.4% in the general population following diabetes and thyroid disorders [1]. However, primary hyperparathyroidism in pregnancy is rare [2]. The incidence of primary hyperparathyroidism in pregnancy is around 8 cases annually per 100,000 of population [2].

Several studies and reports published in the literature showed that there is a possible association between primary hyperparathyroidism and preeclampsia. A recent study done by Hultin et al. showed that women with primary hyperparathyroidism are 6 times more likely to develop preeclampsia [3]. Another review of 16 primary hyperparathyroidism cases concluded that 25% is the occurrence of preeclampsia among studied women [4]. In this paper, we report a case of primary hyperparathyroidism and preeclampsia demonstrating the yield of the surgical intervention in such cases.

## 2. Case

A 28-year-old woman (gravida 2, para 0, and abortus 1) at 27th week of gestation was referred from a general practitioner's office to the emergency department (ER) of a tertiary care hospital (King Khalid National Guard Hospital, Jeddah, Saudi Arabia) owing to an incidental finding of elevated blood pressure (BP) levels (194/115 mmHg). The patient was managed as a case of preeclampsia. She received MgSO_4_ (a 4 g loading dose, with 1 g/h administered IV thereafter) and dexamethasone (6 mg administered IM every 12 h for a total of 48 h). At that time, she did not complain of any signs or symptoms indicative of preeclampsia. Hence, her diagnosis shifted to gestational hypertension. She was subsequently transferred to the antenatal care ward for observation and further workup. The patient was started on a low-salt diet. MgSO_4_ therapy was discontinued, and she was managed with 200 mg of labetalol administered twice daily.

**Figure 1:**
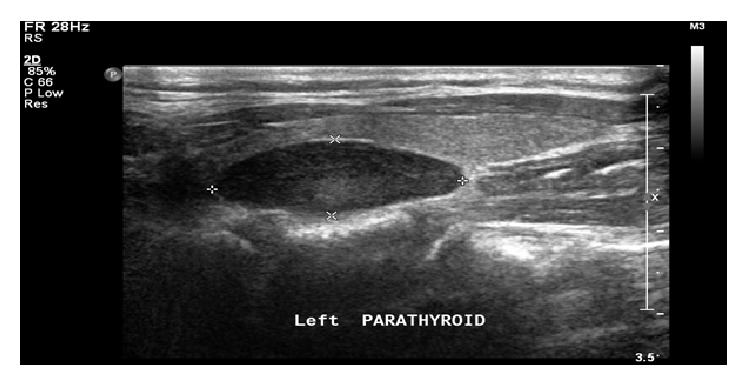
A well-defined, oval shaped, hyperechoic lesion identified in the inferior aspect of the left thyroid lobe exhibits mild-to-moderate internal vascularity on Doppler interrogation. It measures about 2.3 × 1 × 0.8 cm suggestive of parathyroid adenoma.

The day after admission, an elevated adjusted calcium level (2.97 mmol/L) was incidentally detected on routine laboratory results (reference range, 2.1–2.55 mmol/L). Further workup revealed an elevated serum parathyroid hormone (PTH) level (296 pg/mL, reference range 24–114 pg/mL). Ultrasonographic examination of the neck revealed the presence of left inferior parathyroid adenoma (Figure 1). She was diagnosed with primary hyperparathyroidism and treatment was initiated with 125 mL/hour of intravenous normal saline. All calcium supplements were discontinued. Consultation was obtained from otolaryngology team, and surgical resection of the adenoma was scheduled.

On the sixth day of hospitalization, the patient complained of headache and epigastric pain. Her BP was 141/87 mmHg, temperature 37.2 Celsius, pulse 85 beats per minute, and respiratory rate 18 breaths per minute with normal oxygen saturation on room air (97%). 24-hour urine collection revealed 827 mg of protein. Complete blood count, liver and kidney function tests, and coagulation profile were all within normal limits. The patient was diagnosed as preeclampsia with severe features. She was then transferred to the labor and delivery room and given intravenous MgSO_4_ based on the standard protocol. Fetal nonstress test was found to be “nonreassuring.” Subsequently, a decision was made to perform an emergency cesarean section and partial inferior parathyroidectomy.

She delivered a male infant with birth weight of 840 g, head circumference of 24 cm (10th percentile), and length of 32 cm (3rd percentile), consistent with asymmetric intrauterine growth restriction (IUGR). The neonate was then transferred to the NICU. He suffered from jaundice of prematurity. Clinical management in the NICU was generally well tolerated, and a 60-day stay was anticipated. The baby was normocalcemic with no signs of tetany. Postoperatively, the mother had a normal serum PTH (58 pg/mL) and adjusted calcium levels (2.50 mmol/L). The patient was discharged three days later and was scheduled for short-term outpatient followup.

## 3. Discussion

Primary hyperparathyroidism is defined as an elevated PTH level caused by increased production from an adenoma, benign tumor, or malignant tumor of the parathyroid glands. Primary hyperparathyroidism can present with a wide range of symptoms from nonspecific discomfort (e.g., nausea, vomiting, and fatigue) to end-organ damage [5, 6]. Though primary hyperparathyroidism is a relatively common disorder in the general population, it is considered rare in pregnancy [1, 2]. Primary hyperparathyroidism diagnosis can be challenging during pregnancy since increased calcium levels might be masked [5].

Preeclampsia is defined as new-onset of high BP and proteinuria after 20 weeks of pregnancy. Clinically, women with preeclampsia present with a variety of signs and symptoms such as generalized edema, headache, visual disturbance, and abdominal pain [3]. It is suggested that primary hyperparathyroidism and preeclampsia are independent and unrelated disorders [7]. On the other hand, others proposed that they are closely related and that preeclampsia might persist after delivery among patients with primary hyperparathyroidism [3–5]. The link between hyperparathyroidism and hypertensive disorders is proposed to be related to the interaction of PTH with the renin-aldosterone system, the sympathetic nervous system, and the vascular endothelium [5, 7].

Severe hypertension and preeclampsia in patients with primary hyperparathyroidism can lead to severe complications, including intracerebral hemorrhage, retinal hemorrhage, parathyroid hemorrhage, and hypertensive crisis [5, 7]. However, published data have reported favorable maternal outcomes with prompt diagnosis and management of hyperparathyroidism and preeclampsia [5–7]. Fetal or neonatal outcomes are highly variable and dependent on the maternal condition, the week of gestation, the degree of hypocalcemia, and the availability of specialist intensive care units [8–11].

Currently, there are no guidelines available for the treatment of primary hyperparathyroidism during pregnancy [12]. Therefore, the treatment plan should be individualized depending on the symptoms, the estimated risk to the health of both the mother and her fetus, and the gestational age [6]. Conservative options and pharmacological agents include rehydration, calcitonin (pregnancy category C), bisphosphonates (pregnancy category D), and cinacalcet (pregnancy category C). However, similar to the nonpregnant population, surgical parathyroidectomy is the only cure [4, 6]. The optimum timing of surgery during pregnancy is not rigid; however, intervention during the second trimester is preferable [6].

This patient presented in the early weeks of the third trimester of pregnancy and this is consistent with other cases reported in the literature [8–11]. She had an insidious onset of preeclampsia following hyperparathyroidism. Other reported cases presented initially with nausea, vomiting, and hyperemesis [7], abdominal pain [5], headache [11], and hypertension [8–11]. Postpartum course of the mother went smooth and uneventful after appropriate management by the treating multidisciplinary team. The baby condition was complicated by low birth weight and asymmetrical IUGR which was expected from the overall pregnancy circumstances as in other reports [8–10]. Hence, primary hyperparathyroidism poses a risk for preterm delivery.

## 4. Conclusion

The association between primary hyperparathyroidism and preeclampsia is increasingly being recognized. We are reporting this case to increase the awareness of healthcare providers who take care of pregnant ladies about the possibility that primary hyperparathyroidism could be a risk factor for preeclampsia. Further research is needed to discover the pathophysiology, possible presentations, appropriate management, and prevention of such cases.
